# ASSESSING PHYSICAL FUNCTION AND MENTAL HEALTH AMONG OLDER PERSONS IN PRISON

**DOI:** 10.1093/geroni/igac059.1682

**Published:** 2022-12-20

**Authors:** Lisa Barry

**Affiliations:** University of Connecticut Center on Aging, Farmington, Connecticut, United States

## Abstract

As the number of older incarcerated persons grows, evaluating changes in their physical and mental health over time may be important for appropriate planning and needs assessment. Based on findings from the Aging Inmates’ Suicidal Ideation and Depression Study (Aging INSIDE), we will provide recommendations for assessing both objective and subjective physical function in older incarcerated persons. We will also discuss our experiences with assessing depression and suicidal ideation in this population and describe how the processes for collecting data needed to be modified during the COVID-19 pandemic. Those attending this session will learn about the pros and cons of using face-to-face assessments and mailed surveys to assess physical function and mental health among older persons in the prison setting and will learn how these outcomes may differ between those who have a life sentence versus those expecting to be released from incarceration in late life.

